# Online Learning-Related Visual Function Impairment During and After the COVID-19 Pandemic

**DOI:** 10.3389/fpubh.2021.645971

**Published:** 2021-11-29

**Authors:** Qian Fan, Hongxia Wang, Wenjun Kong, Wei Zhang, Zhouyue Li, Yan Wang

**Affiliations:** ^1^Tianjin Key Lab of Ophthalmology and Visual Science, Tianjin Eye Hospital and Eye Institute, Nankai University Affiliated Eye Hospital, Clinical College of Ophthalmology Tianjin Medical University, Tianjin, China; ^2^Shanghai Guanghua Integrated Traditional Chinese and Western Medicine Hospital, Guanghua Hospital Affiliated to Shanghai University of Traditional Chinese Medicine, Shanghai, China; ^3^Beijing You'an Hosptial, Capital Medical University, Beijing, China; ^4^State Key Laboratory of Ophthalmology, Zhongshan Ophthalmic Center, Sun Yat-sen University, Guangzhou, China

**Keywords:** COVID-19, online learning, visual function, pandemic, measures

## Abstract

This study aimed to review the consequences of increased online learning, which was precipitated by the coronavirus disease 2019 (COVID-19), on visual function, as well as the methods for preventing the associated visual impairment. The recent finding implies that a higher incidence of myopia may be observed during the pandemic than that before. The myopia prevalence was 59.35% in COVID-19, which was higher than that in the normal period. COVID-19-related influence of developing myopia among students should be addressed and under control. Online learning precipitated by COVID-19 is likely to increase the global burden of visual function impairment. This review highlighted useful measures to prevent online learning-related visual function impairments, including the following: (1) desktop illumination of no >300 lx, online learning time for primary, and middle-school students of no more than 20–30 min *per session*; (2) daily video time for preschool children not exceeding 1 h, and for school-age children and adolescents not exceeding 2 h; (3) after every 30–40 min of online learning, moving eyes away from the screen or closed for 10 min; (4) engaging in outdoor activities for ≥ 2 h a day; (5) suitable screen and learning environment settings and correct postures for reading and writing; (6) sufficient sleep and proper nutrition. Preventing online learning-related visual impairment during and after this unprecedented pandemic will facilitate future ophthalmic practice.

## Introduction

The coronavirus disease 2019 (COVID-19) has spread worldwide, and the pandemic poses a global threat to public health (1). Aggressive government policies had been implemented to combat the pandemic, and strict measures were implemented regarding social interactions, travel, workplace distancing, and school closures at the beginning of the COVID-19. The United Nations Educational, Scientific and Cultural Organization (UNESCO) says more than 160 countries have closed schools to prevent the spread of COVID-19, which accounts for 87% of the student population around the world (2). More than 180 million primary and secondary students and 47 million preschool children were asked to choose home confinement to reduce the spread of COVID-19 (3). Various universities and schools have announced postponed previously planned re-openings (4, 5). The web-based online learning model has become the most convenient and effective way for students to gain new knowledge and ensure the progress of their learning (6, 7). Most students at all levels have successfully attended web-based distance educational activities such as online teaching *via* web-based video display terminals (VDTs) (8). At Cambridge University, for instance, most online teaching and learning sessions are conducted *via* Zoom (9). Students at both a distant campus and the main campus reported improvements in knowledge (10). However, web-based online learning at home is associated with a longer screen time than campus learning, which may lead to a decrease in outdoor activities, irregular sleep patterns, and less favorable diets. Firstly, increased screen time, near work, and limited outdoor activities were all found to be associated with the onset and progression of myopia and could potentially be aggravated during and post the COVID-19 pandemic (11). The COVID-19 pandemic has also changed the learning modalities, which have consequential behavioral and health implications for youth (12, 13). The teaching model has transferred from school to family and from offline to online amidst COVID-19. Evidence suggests that when children are out of school, they are physically less active, have much longer screen time, irregular sleep patterns, and have less favorable diets, resulting in weight gain and a loss of cardiorespiratory fitness (14, 15). At the same time, some evidence support that the increased e-learning by digital screens and reduction in outdoor activities are two likely channels posing adverse risks for myopia development (16). In most countries it is projected that medium to long-term social distancing measures may curtail outdoor activities, leading to the undesirable effect of more time spent indoors on recreational digital screen time. In another online survey, the association between digital screen use, outdoor activity, lighting condition, and myopia development among school-age children in China during the COVID-19 were investigated (17). The differences in the effects of various modes and time of VDTs on vision are absolutely obvious in the myopia progression during COVID-19. Articles have been published recently to investigate and evaluate the impact of home confinement on the progression of myopia among students in China (18, 19). One survey was conducted by enrolling 1,733 and 1,728 students in 2020 and 2019 in Chongqing, respectively (16). It found that the percentage of myopic students increased by 10.40% during the COVID-19 (16). The study of Wang et al. investigated the prevalence of myopia in the 6–8 age group, which found the prevalence was 21.5% at 6 years, 26.2% at 7 years, and 37.2% at 8 years in the COVID-19 (18). VDTs could be divided into iPhone, computer, TV, and so on. On the one hand, different types of VDTs used for online studies were related to myopic progression. Most students mainly used mobile phones and computers for studying, which had worse uncorrected visual acuity (UCVA) and spherical equivalent (SE) than those who used TV. The average duration of online classes, the number of online classes per day, as well as digital screen exposure time, were negatively correlated with SE, and the average time of outdoor activity was positively correlated with SE. Increased digital screen exposure contributes to myopic progression in students of Chongqing. On the other hand, paper homework and computer homework were both belong to near-distance work. The study of 5,074 children in Rotterdam, found an association between increased computer use and myopia (20). The combined effect of near work, including computer use, reading time, and reading distance, increased the odds of myopia (20). There is also substantial evidence supporting the recommendation that near work causes myopia (21, 22). Therefore, suitable digital devices should be provided for online classes, less near work, and more outdoor activity should be advocated to prevent myopic changes during the COVID-19 pandemic. 1,001,749 students among schoolchildren in 1,305 elementary and high schools in 11 districts of Wenzhou were included to assess the impact of COVID-19 quarantine on myopia progression and incidence. 12,013 students were randomly collected their outdoor time and online learning time during the normal period and COVID-19 quarantine. The myopia prevalence in the total sample was 59.35% in COVID-19, which is higher than that in the normal period. COVID-19-induced influence of developing myopia among students should be addressed and under control (19).

All of these changes, which could potentially aggravate during and after the COVID-19 pandemic, are associated with the onset and progression of myopia and may affect the visual health of students (11, 11, 23–26). Based on the latest survey of 14,532 students from nine Chinese provinces conducted by the Chinese Ministry of Education, the incidence of myopia has increased by 11.7%, compared with that at the end of 2019. In particular, the incidence of myopia in primary school students has increased by 15.2%; that in junior high school students has increased by 8.2%; and that in high school students has increased by 3.8% (27). The incidence of myopia was 45.8% for students who took online classes for >1 h every day, and 76.7% for those who took online classes for more than 4 h daily. From January to July 2020, the average daily effective outdoor exposure time for young people was seriously insufficient, specifically only 32.3 min, which does not even reach one-third of the recommended value (27). During the COVID-19 pandemic, the average reading environment illumination was only 109.7 lx, which was lower than the recommended value (125 lx) (27). In the latest study, outdoor activity time was significantly negatively associated with increased myopia incidence during 6-month before and after the COVID-19. During the COVID-19, the outdoor activity times decreased by 1.14 h in Grades 1–6 and 1.71 h in Grades 7–12 (19). The study of Ma et al. reported that children were at higher risk of myopia progression during COVID-19, which was associated with longtime online learning and digital screen reading (25).

In light of the abovementioned data, the national guidelines for preventing and controlling myopia in children and adolescents during home quarantine of infectious disease epidemic were released on July 28, 2020 (28). Therefore, it is of utmost importance to understand, in further detail, the online learning-related factors that affect ocular health, to implement effective measures to protect the visual function and to prevent the development of eye diseases in the future.

## Possible Effects of Web-Based Online Learning on Visual Function

Recent research has reviewed the practices related to visual impairment and myopia during the COVID-19 pandemic from Asian to European nations (11, 23, 24). This pandemic has compromised normal school teaching and will likely have long-lasting effects in this area. During the pandemic, outdoor sports time for students has decreased sharply and the daily web-based online learning time might even reach up to 12 h in some cases (8, 23). Some changes in learning patterns that are evident include an increased burden of intensive education, prolonged near-work activities, and limited time spent outdoors. All of these constitutional changes will sustain after the re-opening of schools during and after the COVID-19 pandemic. Except for genetic susceptibility, environmental changes, and cultural exposure are predominant factors for the occurrence and development of visual function impairments, which have exacerbated during the present pandemic.

### Visual Fatigue

Visual fatigue can be caused by accommodative dysfunctions. Devices with small screen sizes such as iPads and smartphones, which are used in digital teaching, tend to display crowded fonts and reduce row spacing while offering limited display brightness; this generates the need for more eye adjustment and radial movement (29, 30). Overuse of VDTs may cause an overall feeling of discomfort and body aches and may contribute to the development of ophthalmic diseases (29–31). The more the use of VDTs, the more the risk of visual fatigue. Visual fatigue can progress to myopia, esotropia, and other ophthalmic diseases. Visual fatigue could be caused by excessive application of near vision, which was common after a long time VDTs usage. Visual fatigue can alter the refractive state in a partially reversible manner. Chang et al. reported accelerated myopic progression during the COVID-19 pandemic lockdown in children and teenagers, which was partially reversed after lockdown, suggesting that both accommodative spasm and structural changes contributed to this accelerated rate (32). However, digital eye strain is a result of the current restriction measures that have led to an imposed home confinement (33). Visual fatigue can also induce acute acquired concomitant esotropia, which was reported during the COVID-19 pandemic (34).

### Myopia Development

The global restrictions imposed due to COVID-19 have severely limited the hours spent outdoors and, consequently, reduced the exposure to sunlight and the exercises for far vision. Online courses, games, storybooks, and entertainment videos, among others, constitute a forced adaptation of digital teaching over face-to-face teaching methods (35). If visual fatigue is not relieved promptly, it can further progress into myopia after prolonged online learning. Distance vision fluctuations, squinting when gazing at distant objects, and other symptoms are occasionally seen in myopic students. The study of Chen et al. reported that compared to the pre-COVID-19 period, a higher incidence of myopia was likely to be observed in China, where schools have replaced books with tablets and computers (36). The work of Bez et al. reported that intensive near-work activities played a part in the occurrence of myopia (37). The study of Xu et al. found that online time of students was significantly positively associated with increased myopia incidence (19). Liu et al. reported that remote learning-induced extended duration of daily digital screen usage, and the risks of myopic symptom onset and progression increase during the outbreak of COVID-19 (38). In particular, myopia can induce serious sight-threatening ocular diseases, including glaucoma, cataract, myopic retinal degeneration, retinal detachment, and even blindness (39).

### Dry Eye Syndrome

Previous studies have shown that blinking frequency can be reduced by nearly 65% when the sight of an individual is fixed on VDTs for more than 30 min (40). During online learning, long-term attention to the screen can reduce the blinking frequency and tear film renewal (41). Blue light from electronic screens can reduce tear film stability, thereby leading to eye discomfort and dry eye, which can manifest as dryness sensations, burning sensations, foreign body sensations, photophobia, vision fluctuations, etc., (42). The study of Xu et al. reported that usage of VDTs can lower the stability of tear film. High-energy blue light from VDTs can be a risk factor in the ocular surface damage, but the damage is reversible (43). The work of Kaido et al. found that the blue light exposure might be harmful to visual function in patients with short tear break-up time and dry eye. Protecting the eyes from short-wavelength blue light may help to ameliorate visual impairment associated with tear instability in patients with dry eye (44). Modern light sources like LEDs and displays tend to emit blue light. The effect of blue light on the retina is called blue light hazard and is studied extensively. In an animal model, all investigated irradiation sources cause cataracts in porcine lenses-even blue visible light (45). There is a sense in which electronics are more convenient than books in effective online learning and ensure that the contents of the courses meet the educational requirements. Yet it is also important not to neglect its side effects, which include higher screen brightness induced eye strain and cataract possibility.

In addition, during the COVID-19 pandemic, long-time displacement or incorrect fitting of the face mask could disperse air around the eyes, increase ocular irritation and tear evaporation, with effects similar to those experienced by users of continuous positive airway pressure machines (46, 47). Dry eye syndrome might therefore be more common in the COVID-19 era than before (47, 48).

### Irregular Astigmatism

An unstable tear film in dry eyes caused by a long-term fixation on VDTs can increase the likelihood of decreased vision, blurred vision, ghosting, compensatory head position, and squinting vision (49). Koh found increases in irregular astigmatism and higher-order aberrations in the eyes of patients with dry eye syndrome. According to the Fourier harmonic analysis, the amount of corneal irregular astigmatism was significantly lower in normal eyes than in dry eyes (50).

### Acute Concomitant Esotropia

Acute concomitant esotropia mainly occurs in older children and adults. Long-term close-distance work can induce over-regulatory aggregation of both eyes and lead to morbidity, which is manifested as sudden esotropia or esotropia with diplopia and binocular vision (51). Intense near work can also induce acute acquired concomitant esotropia, which was reported during the COVID-19 pandemic (34).

### Others

In addition to the above diseases, long-term screen time of web-based online learning and lack of physical activities may also lead to anxiety, depression, and other physical and mental diseases, and maybe related to an increased risk of fundus diseases, such as transient amaurosis, and other ischemic retinopathy (52). Liu et al. found that psychosocial stress accompanying prolonged social isolation during the pandemic was a risk factor for myopia development (53). Lack of social interaction during a pandemic and increasing psychosocial stressors are both risk factors. Xu et al. reported that COVID-19 quarantine-related behavioral changes had an effect on myopia progression among children and adolescents (19).

## Visual Protection Measures During Online Learning

Based on the occurrence and development of some of the abovementioned diseases, which are related to poor eye-use habits and the environment, the following suggestions are proposed to minimize the damage to the visual function of students engaging in online learning (54).

### Screen Settings

When engaging in online learning, large video terminals, such as desktop or portable computer displays, are preferred over smaller screens. Moreover, for the same screen size, choosing electronic products with high screen resolutions is advisable. It is recommended to place the screen of large video terminals more than 50 cm away from the eye and, in the case of smartphones, at no >40 cm away, perpendicular to the line of sight. Electronic products should be placed away from windows and direct light. The height of the screen vertex should be parallel or slightly lower than the eye horizontal line so that the direction of eye gaze is horizontal or slightly lower. The brightness of the screen should not be too high; it should be close to or slightly higher than the brightness of the surroundings. The font on the screen should be large enough for comfortable viewing (28).

### Illumination and Learning Environment Settings

The intensity of outdoor sunlight is hundreds of times higher than that of indoor lighting. The stronger the light, the greater the release of dopamine, which may participate in the occurrence and development of myopia (55). The leading hypothesis is that light stimulates the release of dopamine in the retina, and the neurotransmitter, in turn, blocks the axial length elongation (56). Additionally, high-intensity light can shrink pupils, deepen the depth of field, and reduce blurring, which may also suppress myopia (57, 58). However, the strength of illuminance in a well-lit classroom is generally no more than 500 lx. Dolgin et al. estimate that children need to spend around 3 h per day under light levels of at least 10,000 lx to be protected against myopia (56). Following the abovementioned study, Yang et al. collected the changes of eyes and analyzed the risk factors on near-work and outdoor activities using Clouclip devices. A light intensity of >3,000 lx for outdoor activities was protective for myopic and non-myopic children (58). Under these circumstances, during the daytime, students should make full use of the natural light when studying at home. If the natural light is too strong during the daytime, curtains should be used for shading, and they should be adjusted according to the angle of sunlight (28). Lamps with higher illuminance should be used as lighting choices (28). The average illumination of the desktop should be no <300 lx. For reading and writing at night, it is advisable to use the room ceiling lamp and the desk lamp at the same time, and the latter should be placed in front of the students. When using a white light-emitting diode (LED) desk lamp with adjustable color temperature, the color temperature should be adjusted below 4,000 K (Kelvin; color temperature unit) at night; LED desk lamps with non-adjustable color temperatures and with color temperatures higher than 4,000 K should not be used at night. It is not advisable to use naked lamps without lampshades or to place glass plates or other articles that are likely to produce glares on the desk (28). Proper learning environment settings and the use of a high light intensity could help to prevent myopia. On the other side, as the eyes of humans are more and more exposed to these devices (e.g., LED lights, iPads, mobile phones, computers, and other devices), excessive blue light radiation may induce dry eyes, fundus lesions (60). However, the specific cause and effect between blue light and myopia development are not clear. The effect of blue light illuminance needs additional studies in the future.

### Posture Requirements for Reading and Writing

Learning postures at home may be very casual, for instance lying down or sitting anywhere. Near distance, reading is much more common during home quarantine. Non-standard postures and low reading distances may significantly increase the chance of visual fatigue and myopia. Wen et al. found that prolonged exposure to screens within a distance of <20 cm was a risk factor for myopic change (58). The study of Ip et al. also indicated that a working distance of <30 cm could independently increase the chance of myopia (61). In addition, the Anyang Childhood Eye Study reported that the reading distance of ≤ 20 cm was significantly associated with axial length (62). Therefore, maintaining a proper distance is crucial when reading and writing. The distance between eyes and books should be approximately one foot (33 cm), the distance between chest and desk should be approximately one fist (6–7 cm), and the distance between the finger holding the pen and the tip of the pen should be approximately one inch (3 cm) (63). When viewing electronic devices, the head and neck should be kept straight or slightly tilted forward to avoid head and neck side deviation, excessive forward extension, backward tilt, or excessive bow (28).

### Adequate Eye Rest

The guidelines suggest that the online learning time for primary school students should be no more than 20 min, and for middle school students, no more than 30 min per session. The daily video time for preschool children should not exceed 1 h, and that for school-age children and adolescents should not exceed 2 h. The younger the child, the less time they should spend viewing a screen (28). After every 30–40 min of learning, the eyes should be rested for 10 min; it is recommended to look far away from the screen or close the eyes to relax the ciliary muscle. In addition, resting reduces unnecessary screen time. The use of electronics for non-learning purposes should not exceed 15 min per session among children (63).

### Outdoor Activities

Inadequate outdoors time has been recognized as an important risk factor for myopia (64–66); recent reports suggest that adequate time spent outdoors may help prevent myopia (65, 66). Exposure to natural light for several hours a day has been shown to suppress abnormal axial growth of the eye (67). Light intensity plays an important role in the protective effect of outdoor time (68). The study of Wu et al. found that prolonged exposure to moderate light intensities, i.e., 1,000 lx or more (vs. 3,000 lx or more), may have a similar effect (67). The work of Morgan et al. estimated that approximately 3 h per day under light levels of at least 10,000 lx was essential for myopia prevention (59). To prevent myopia shift and progression, in the elementary school system, children should be encouraged to spend enough outdoor time every week both at school and outside of school (69). Increasing outdoor activities helped prevent myopia occurrence and progression, as well as axial eye growth in children (70). Increased outdoor activities are proven to be protective factors for myopia, as near-distance work and higher education levels affect the opposite (71).

Therefore, during the pandemic, exercising and relaxing under sunshine for more than 2 h every day is recommended for adequate exposure to outdoor natural light. Examples of suitable activities include body exercises, skipping rope, sit-ups, and push-ups (28, 63). In addition, more outdoor time is recommended post-pandemic.

### Adequate Sleep and Nutrition

In one large cross-sectional study on children and adolescents, myopia was associated with shorter self-reported sleep duration (72). Sufficient sleep can effectively relax the ciliary muscle and relieve visual fatigue. Primary school students, junior and senior high school students sleep for more than 10, 9, and 8 h a day, respectively (28, 63). Irregular sleep patterns may affect circadian rhythms which may be involved in the development of myopia. In addition, omega-3 unsaturated fatty acids, carotenoids, as well as vitamins A, C, D, and E can improve dry eye syndrome and other eye diseases. Eating salmon and other fish rich in omega-3 unsaturated fatty acids, dark green leafy vegetables, and fruits, among others, can ensure an adequate intake of the abovementioned nutrients (73).

### Ophthalmic Consultation and Guidance on Eye Health

Prevention is the main strategy for visual impairment management during and after the pandemic. Ensuring an ergonomic work environment, frequent visual examinations, and eye care are of utmost importance to treat visual disorders. Online consultation, intelligent detection platforms, and home inspection equipment can be used to preliminarily evaluate visual function. In cases of sudden visual acuity decline, severe eye pain, photophobia, or tears, patients should immediately go to the ophthalmic emergency department for prompt medical treatment. Visual impairment changes may be detrimental to the mental health of students, which reminds us that psychological consultation should not be ignored (74).

This study has certain limitations, mainly resulting from the unprecedented nature of and the global uncertainty regarding the pandemic, as well as from the short time available for collecting, processing, and publishing data and evidence from large samples.

## Conclusion

The COVID-19 pandemic has led to dramatic changes in many aspects of daily life, including less physical activity, prolonged screen time, irregular sleep patterns, and psychological changes (24). Large-scale web-based online learning has become the mainstream public learning mode during the pandemic. This article analyzed and provided measures to prevent web-based online learning-related visual function impairment. Will online teaching lead to an increased incidence of myopia and its progression in students with pre-existing myopia? These questions are likely to be answered in future studies. After the initial peak of COVID-19, and even with the highly effective medical interventions developed, cities worldwide will likely experience lingering outbreaks of the disease for months and, potentially, for years. When and how the pandemic will end remains uncertain. Therefore, various types of measures have been suggested to prevent the incidence and progression of visual impairment. Suitable screen and learning environment settings, correct postures for reading and writing, proper rest and sufficient outdoor activities, and sufficient sleep and proper nutrition are all essential to prevent visual impairment and to control pre-existing visual conditions during and after the pandemic (28, 63, 73, 74). Being a convenient and efficient way of learning, online learning is expected to become an important tool for the public to continuously acquire knowledge during and after the pandemic. Children, teenagers, and even adults should pay attention to eye care habits while making full use of online learning resources. In addition, online teaching should be planned scientifically, including reasonable scheduling of online classes and increasing eyesight protection. Knowledge regarding protective measures for visual function and increased awareness of visual health among ophthalmic consultations should be used to avoid damage to visual function and eye diseases caused by inappropriate online learning methods. It is necessary to emphasize the prevention of web-based online learning-related visual impairment during and after this unprecedented pandemic, which will contribute to future ophthalmic practice.

## Author Contributions

QF, HW, and WK designed and finished analysis, programming, and writing this article. WZ and ZL analyzed and gave comprehensive feedback on the manuscript. YW addressed all the feedback and finalized this manuscript. All authors have read and agreed to the published version of the manuscript.

## Funding

This project was supported by the National Natural Science Foundation of China (YW: 81670884 and 81873684) and the Key Projects of Science and Technology Fund of Tianjin Health and Family Planning Commission (QF: 2014KR17), the Key Project of Tianjin Eye Hospital (QF: YKYB2007).

## Conflict of Interest

The authors declare that the research was conducted in the absence of any commercial or financial relationships that could be construed as a potential conflict of interest. The handling editor declared a shared affiliation, with one of the authors WK at the time of the review (2977855).

## Publisher's Note

All claims expressed in this article are solely those of the authors and do not necessarily represent those of their affiliated organizations, or those of the publisher, the editors and the reviewers. Any product that may be evaluated in this article, or claim that may be made by its manufacturer, is not guaranteed or endorsed by the publisher.

## Abbreviations

COVID-19: coronavirus disease 2019
VDTs: video display terminals
UNESCO, United Nations Educational: Scientific and Cultural Organization
LED: light-emitting diode.
